# Sequence feature-based prediction of protein stability changes upon amino acid substitutions

**DOI:** 10.1186/1471-2164-11-S2-S5

**Published:** 2010-11-02

**Authors:** Shaolei Teng, Anand K Srivastava, Liangjiang Wang

**Affiliations:** 1 Department of Genetics and Biochemistry, Clemson University, Clemson, SC 29634, USA; 2 J.C. Self Research Institute of Human Genetics, Greenwood Genetic Center, Greenwood, SC 29646, USA

## Abstract

**Background:**

Protein destabilization is a common mechanism by which amino acid substitutions cause human diseases. Although several machine learning methods have been reported for predicting protein stability changes upon amino acid substitutions, the previous studies did not utilize relevant sequence features representing biological knowledge for classifier construction.

**Results:**

In this study, a new machine learning method has been developed for sequence feature-based prediction of protein stability changes upon amino acid substitutions. Support vector machines were trained with data from experimental studies on the free energy change of protein stability upon mutations. To construct accurate classifiers, twenty sequence features were examined for input vector encoding. It was shown that classifier performance varied significantly by using different sequence features. The most accurate classifier in this study was constructed using a combination of six sequence features. This classifier achieved an overall accuracy of 84.59% with 70.29% sensitivity and 90.98% specificity.

**Conclusions:**

Relevant sequence features can be used to accurately predict protein stability changes upon amino acid substitutions. Predictive results at this level of accuracy may provide useful information to distinguish between deleterious and tolerant alterations in disease candidate genes. To make the classifier accessible to the genetics research community, we have developed a new web server, called MuStab (http://bioinfo.ggc.org/mustab/).

## Background

Amino acid substitutions can cause a series of changes to normal protein function, such as geometric constraint changes, physico-chemical effects, and disruption of salt bridges or hydrogen bonds [1] . These changes may lead to protein destabilization or some abnormal biological functions. Previous studies suggest that each person may have 24,000 – 40,000 non-synonymous Single Nucleotide Polymorphisms (nsSNPs), and there are a total of 67,000 – 200,000 common nsSNPs in the human population [2] . These nsSNPs give rise to amino acid substitutions in proteins. While most nsSNPs appear to be functionally neutral, the others affect protein function and may cause or influence diseases. Yue and Moult [3] investigated the effect of amino acid substitutions on protein stability, and estimated that approximately 25% of nsSNPs in the human population might be deleterious to protein function. Of the known disease-causing missense mutations, the vast majority (up to 80%) resulted in protein destabilization [4] . However, it is not feasible to experimentally determine the effect of each human nsSNP on protein stability. Rather, computational methods are needed to provide fast and efficient tools for examining a large number of nsSNPs for potential disease-causing mutations.

Machine learning has been applied to sequence-based prediction of protein stability changes upon amino acid substitutions [5][6][7][8] . The machine learning problem can be specified as follows: given the amino acid sequence of a protein and a single amino acid substitution, the task is to predict whether the substitution may alter protein stability. By using the available data from experimental studies, classifiers can be constructed for predicting either the free energy change (ΔΔ*G*) of protein stability upon mutations or the direction of the change (increased stability if ΔΔ*G >* 0, or decreased stability if ΔΔ*G* < 0). Nevertheless, for many biological applications, correctly predicting the direction of the stability change (a binary classification problem) is more relevant than estimating the magnitude of the free energy change (a regression problem) [5] .

Capriotti et al. [5] reported an artificial neural network-based method for predicting the direction of protein stability changes upon point mutations. The predictor was trained with protein sequence alone. It was shown that the sequence-based system could be used to complement the available energy-based methods for improving protein design strategies. The same research group also developed support vector machine (SVM) models for sequence- based prediction of both the free energy change and the direction of the change upon mutations [6] . These SVM models were used to develop the I-Mutant2.0 web server, which could predict protein stability increase or decrease at the overall accuracy of 77% (based on cross-validation). Interestingly, it was found that the sequence-based system was almost as accurate as the structure-based method (80% overall accuracy) on the same dataset [6] . This observation was further confirmed by Cheng and coworkers, who trained SVMs for predicting protein stability changes from amino acid sequence and structural information [7] . More recently, Huang and coworkers developed the iPTREE-STAB web server, which used decision trees with an adaptive boosting algorithm to discriminate stabilizing and destabilizing substitutions in protein sequences [8] . Among all the existing methods, iPTREE-STAB achieved the best classifier performance in cross-validation tests (82.1% overall accuracy with 75.3% sensitivity and 84.5% specificity).

The above-mentioned studies suggest that protein stability changes can be predicted directly from primary sequence data with similar prediction accuracy as structure-based methods. The sequence-based approach is particularly appealing since structural information is still not available for most proteins. However, little domain-specific knowledge in terms of biological features was used for classifier construction in the previous studies [5] . In the present study, we have examined twenty sequence features for classifier construction. Support vector machines (SVMs) have been trained with the feature-encoded data instances of protein stability changes upon amino acid substitutions. Our results indicate that accurate SVM models can be constructed by using relevant sequence features for input vector encoding. To make the classifier publicly available, we have developed a new web server, called MuStab (http://bioinfo.ggc.org/mustab/).

## Methods

### Data

The dataset used in this study was derived from two previous studies [6][8] , in which experimental data for the free energy changes of protein stability upon mutations were collected from the ProTherm database [9] . To construct a robust classifier, data redundancy was removed and the dataset had less than 25% identity among the amino acid sequences. Each data instance in the dataset had the following attributes: amino acid sequence, wide- type amino acid identity and sequence position, mutant amino acid identity, pH value, and free energy change. If the free energy change was negative (protein destabilization), the instance was labelled as a negative example. Otherwise, the instance was labelled as a positive example. The dataset contained 464 positive instances and 1,016 negative instances.

### Sequence features

Twenty sequence features were used to code each amino acid residue in a data instance. The sequence features were obtained from Protscale [10] (http://expasy.org/tools/protscale.html) and AAindex [11] (http://www.genome.jp/aaindex/). These features fall into the following four classes:

**1) Biochemical features:** including molecular weight (feature M); side-chain pK_a_ value (K); hydrophobicity index (H); polarity (P); and overall amino acid composition (Co). Each amino acid has a unique molecular weight (M), which is related to the volume of space that a residue occupies in protein structures. Side-chain pK_a_ (K) is related to the ionization state of a residue, and thus plays a key role in pH-dependent protein stability. Hydrophobicity (H) is important for amino acid side chain packing and protein folding. Hydrophobic interactions make non-polar side chains to pack together inside proteins, and disruption of these interactions may cause protein destabilization. Polarity (P) is the dipole-dipole intermolecular interactions between the positively and negatively charged residues. The amino acid composition (Co) was previously shown to be related to the evolution and stability of small proteins [12] .

**2) Structural features:** including the conformational parameters for alpha-helix (A), beta- sheet (B), and coil (C); average area buried on transfer from standard state to folded protein (Aa); and bulkiness (Bu). Protein secondary structures can be divided into alpha-helix, beta- sheet, and coil conformations. An amino acid often has a different tendency to form one of the three types of secondary structures. For instance, amino acids A, I, E, L and M tend to be in the alpha-helical conformation, whereas K, N and D are often found in beta-sheets. In this study, the conformational parameters reported by Deléage and Roux [13] were used for features A, B and C. Feature Aa is another structural parameter, which estimates a residue’s average area buried in the interior core of a globular protein [14] . Bulkiness (Bu), the ratio of the side chain volume to the length of an amino acid, may affect the local structure of a protein [15] .

**3) Empirical features:** the protein stability scale based on atom-atom potential (S1); the relative protein stability scale derived from mutation experiments (S2); and the side-chain contribution to protein stability (S3). Zhou et al. [16] derived two protein stability scales from atom-atom potential of mean force based on Distance scaled Finite Ideal-gas REference (DFIRE) state (S1) and a large database of mutations (S2). Takano and Yutani [17] calculated the transfer Gibbs energy of mutant proteins, and derived the amino acid scale for the side-chain contribution to protein stability (S3) based on data from protein denaturation experiments.

**4) Other biological features:** including the average flexibility index (F); the mobility of an amino acid on chromatography paper (Mc); the number of codons for an amino acid (No); refractivity (R); recognition factor (Rf); the relative mutability of an amino acid (Rm); and transmembrane tendency (Tt). The average flexibility index of an amino acid (F) was derived from structures of globular proteins [18] . Feature Mc was derived from experimental data by Aboderin [19] . Refractivity (R) refers to protein density and folding characteristics [20] . Recognition factor (Rf) is the average of stabilization energy for an amino acid [21] . The relative mutability (Rm) indicates the probability that a given amino acid can be changed to others during evolution. Feature Tt is the transmembrane tendency scale described by Zhao and London [22] .

### Support vector machine training

Support vector machines (SVMs) are computational algorithms that can learn from training examples for binary classification problems. The SVM learning algorithm can be described by four basic concepts, including the separating hyperplane, the maximum-margin hyperplane, the soft margin, and the kernel function [23] . For a typical linear classifier, a data instance is represented as an *n*-dimensional vector, and an (*n* – 1) dimensional hyperplane is used to separate the positive instances from the negative ones. However, for non-linear classifiers that are generally applicable to biological problems, a kernel function can be used to measure the distance between data points in a higher dimensional space. This allows the SVM algorithm to fit the maximum-margin hyperplane in the transformed space. In this study, we used the radial basis function (RBF) kernel:

     (1)

where  and  are two data vectors, and *γ* is a training parameter. A smaller *γ* value makes decision boundary smoother. The regularization factor C, another parameter for SVM training, controls the tradeoff between low training error and large margin.

The SVMlight software package (available at http://svmlight.joachims.org/) was used to construct the SVM classifiers in this study. Each training instance was a subsequence of *w* consecutive residues, where *w* was also called the window size. The amino acid substitution site was positioned in the middle of the subsequence, and the other (*w* – 1) neighbouring residues provided context information for the substitution site. The input vector was then obtained by encoding each residue with one or more biological features. The input vector also included the pH value at which the free energy change was measured experimentally. In this study, various values of *w*, *γ* and *C* parameters were examined to optimize SVM classifier performance.

### Classifier evaluation

This study used a fivefold cross-validation method to evaluate classifier performance. Positive and negative instances were randomly distributed into five folds. In each of the five iterations, four of the five folds were used to train a classifier, and then the remaining one fold was used as the test data to evaluate the classifier. The predictions made for the test instances in all the five iterations were combined and used to compute the following performance measures:

     (2)

     (3)

     (4)

     (5)

     (6)

where *TP* is the number of true positives; *TN* is the number of true negatives; *FP* is the number of false positives; and *FN* is the number of false negatives. In addition to the commonly used performance measures (overall accuracy, sensitivity and specificity), the average of sensitivity and specificity or the so-called prediction strength [24][25] was also used for classifier comparison in this study. Matthews Correlation Coefficient (MCC) measures the correlation between predictions and the actual class labels. Nevertheless, for imbalanced datasets, different tradeoffs of sensitivity and specificity may give rise to different MCC values for a classifier.

We also used the Receiver Operating Characteristic (ROC) curves [26] for classifier evaluation and comparison. In this study, the ROC curve was generated by varying the output threshold of an SVM classifier and plotting the true positive rate (sensitivity) against the false positive rate (1 – specificity) for each threshold value. Since the ROC curve of an accurate classifier is close to the left-hand and top borders of the plot, the area under the curve (AUC) can be used as a reliable measure of classifier performance [27] . The maximum value of AUC is 1, which indicates a perfect classifier. Weak classifiers and random guessing have AUC values close to 0.5.

## Results and discussion

### Effect of sequence context on classifier performance

We first constructed a classifier using the three biochemical features, including the hydrophobicity index (H), side-chain pKa value (K), and molecular weight (M) of an amino acid. These features were previously selected for DNA and RNA-binding site prediction [24][25] . In the initial attempt to construct a classifier for protein stability prediction, the window size was set to eleven (*w* = 11). Different values of SVM training parameters were tested, and the optimal parameter settings were found to be *γ* = 0.8 and *C* = 1.0. As shown in Table 1, the classifier achieved the overall accuracy (AC) of 81.82% with 74.48% sensitivity (SN) and 85.11% specificity (SP). The prediction strength (ST) reached 79.79% with MCC = 0.5843 and ROC AUC = 0.8804. Therefore, this SVM achieved similar performance measures as the best existing classifier (iPTREE-STAB with 82.1% overall accuracy, 75.3% sensitivity and 84.5% specificity) [8] .

**Table 1:** Effect of window sizes on sequence-based prediction of protein stability changes.

Window size	AC (%)	SN (%)	SP (%)	ST (%)	MCC	ROC AUC
1	66.92	70.69	65.20	67.94	0.3349	0.7425

3	73.91	74.83	73.49	74.16	0.4554	0.7996

5	77.51	76.67	77.90	77.28	0.5194	0.8512

7	80.80	76.43	82.83	79.63	0.5750	0.8737

9	81.28	75.66	83.78	79.72	0.5774	0.8755

11	81.82	74.48	85.11	79.79	0.5843	0.8804

13	82.10	71.84	86.67	79.26	0.5824	0.8797

15	81.45	69.71	86.75	78.23	0.5665	0.8775

17	81.88	69.50	87.58	78.54	0.5779	0.8799

19	81.21	68.80	86.98	77.89	0.5627	0.8779

21	81.29	68.98	86.98	77.98	0.5645	0.8735

To determine whether classifier performance was affected by the sequence context of the substitution site, SVMs were trained with data instances of various window sizes. As shown in Table 1, protein stability prediction was affected by window sizes. The classifier constructed without any context information (*w* = 1) gave 67.94% prediction strength (70.69% sensitivity and 65.20% specificity), MCC = 0.3349 and AUC = 0.7425. The prediction strength, MCC and AUC were improved when neighbouring residues of the substitution site were included for input encoding. The use of *w* = 11 gave the highest prediction strength (79.79%), MCC (0.5843) and AUC (0.8804), and classifier performance was not further improved by including more neighbouring residues (Table 1).

**Figure 1:**
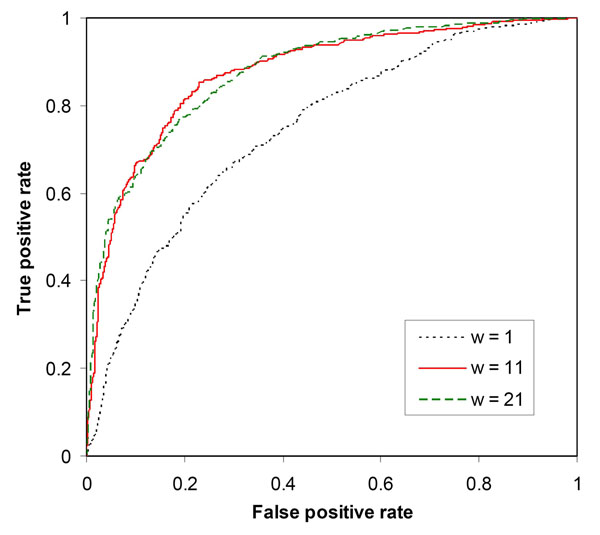
ROC curves to show the effect of context information on prediction of protein stability changes upon amino acid substitutions.

The effect of sequence context information on SVM classifier performance was also demonstrated by using ROC curves. As shown in Figure 1, the ROC curve of the classifier constructed with *w* = 11 was clearly better than the SVM trained without any context information (*w* = 1). However, the use of *w* = 21 did not further improve classifier performance. Thus, eleven residues with the substitution site in the middle position (*w* = 11) appeared to provide enough context information for sequence-based prediction of protein stability changes.

### Relevant sequence features for classifier construction

Many sequence features are available for encoding amino acid residues. To determine which features were relevant for protein stability prediction, we constructed SVM classifiers using each of the twenty sequence features listed in Table 2 for input encoding (*w* = 11). The results were obtained with the training parameters, *γ* = 0.8 and *C* = 1.0. It was found that classifier performance varied significantly by using different features. As shown in Table 2, the highest level of AUC (0.8835) was achieved by using the empirical feature S3 for input encoding. This classifier reached the prediction strength at 79.67% (72.19% sensitivity and 87.15% specificity) and MCC = 0.5922. However, the highest prediction strength at 80.28% (75.62% sensitivity and 84.94% specificity) with MCC = 0.5919 and AUC = 0.8777 was achieved by using amino acid bulkiness (Bu) for input encoding. In contrast, the use of the average flexibility index (F) for input encoding resulted in the lowest prediction strength at 62.02%, MCC = 0.2226 and AUC = 0.6728 (Table 2).

**Table 2:** Predictive performance of classifiers constructed using single sequence features.

Features	AC (%)	SN (%)	SP (%)	ST (%)	MCC	ROC AUC
H	75.88	71.62	77.79	74.70	0.4728	0.8237

K	73.29	73.90	73.02	73.46	0.4402	0.7925

M	68.06	73.52	65.62	69.57	0.3629	0.7480

P	75.94	71.24	78.04	74.64	0.4718	0.8234

Co	70.18	71.62	69.53	70.58	0.3838	0.7586

A	76.41	74.29	77.36	75.82	0.4904	0.8206

B	78.18	74.48	79.83	77.15	0.5199	0.8503

C	72.18	71.05	72.68	71.86	0.4116	0.7847

Aa	79.12	76.57	80.26	78.41	0.5431	0.8459

Bu	82.06	75.62	84.94	80.28	0.5919	0.8777

S1	69.82	70.86	69.36	70.11	0.3756	0.7754

S2	70.24	72.19	69.36	70.78	0.3875	0.7665

S3	82.53	72.19	87.15	79.67	0.5922	0.8835

F	61.41	63.62	60.43	62.02	0.2226	0.6728

R	66.47	65.14	67.06	66.10	0.3008	0.7140

Mc	78.35	73.52	80.51	77.02	0.5202	0.8417

No	69.82	74.86	67.57	71.22	0.3944	0.7656

Rf	62.06	73.71	56.85	65.28	0.2831	0.6889

Rm	75.94	69.90	78.64	74.27	0.4672	0.8118

Tt	83.59	66.48	91.23	78.86	0.6035	0.8704

**Figure 2:**
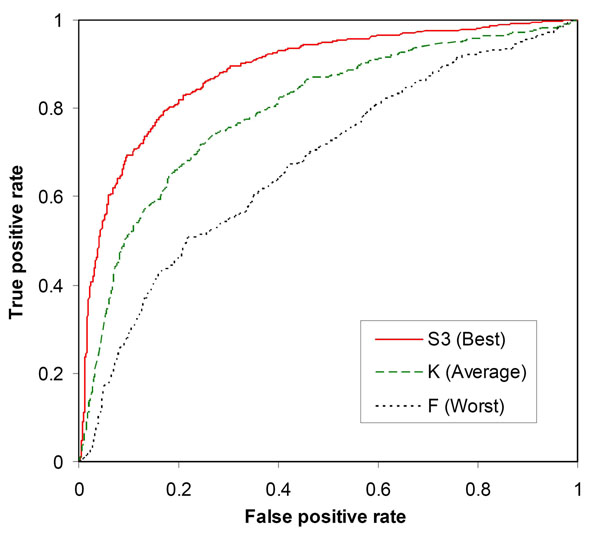
ROC curves to show the different performance levels of classifiers constructed using individual sequence features.

Figure 2 shows the ROC curves of the best and worst classifiers (based on AUC) that were constructed using the individual sequence features. Also shown in Figure 2 is the ROC curve of the SVM classifier constructed with the K feature, which gave approximately the average performance among the sequence features.

The results suggest that a variety of sequence features are relevant for predicting protein stability changes upon amino acid substitutions. Of the five biochemical features (H, K, M, P and Co), the hydrophobicity index (H) gave the best predictive performance at 74.70% prediction strength (71.62% sensitivity and 77.79% specificity), MCC = 0.4728 and AUC = 0.8237 (Table 2). Hydrophobicity is a key factor in amino acid side chain packing and protein folding. Hydrophobicity changes owing to amino acid substitutions may cause proteins not to fold into stable conformation, and thus result in protein destabilization.

Of the structural features (A, B, C, Aa and Bu), bulkiness (Bu) gave rise to the highest prediction strength at 80.28% with MCC = 0.5919 and AUC = 0.8777. In contrast, the classifier using the conformational parameter for coil (*C*) had the relatively low performance with 71.86% prediction strength, MCC = 0.4116 and AUC = 0.7847 (Table 2). The possible explanation is that since coils are often unstructured and flexible, amino acid substitutions in the coil region may not cause significant changes in protein structure and stability.

The empirical features (S1, S2 and S3) are protein stability scales based on experimental data. Interestingly, when used for SVM classifier construction, these features did not give significantly better performance than the other sequence features. While the use of the S3 feature (side-chain contribution to protein stability) resulted in the highest level of AUC (0.8835) with 79.67% prediction strength and MCC = 0.5922, the other two empirical features (S1 and S2) were much less accurate for predicting protein stability changes (Table 2). Thus, it is possible that the empirical features do not capture all the information about the determinants of protein stability.

Of the other biological features, transmembrane tendency (Tt) achieved the highest level of MCC (0.6035) with 78.86% prediction strength and AUC = 0.8704 (Table 2). The feature Mc (the mobility of an amino acid on chromatography paper) also gave rise to relatively high classifier performance (77.02% prediction strength, MCC = 0.5202 and AUC = 0.8417). Therefore, multiple features from each of the four feature classes achieved high performance for predicting protein stability changes upon amino acid substitutions. It might be possible that classifier performance could be further improved by combining several sequence features for input encoding.

### Use of multiple sequence features to improve classifier performance

To examine whether classifier performance could be further improved, we first used all the 20 sequence features for input encoding. Surprisingly, the resulting classifier was not as accurate as some of the SVMs trained with single features (Table 3). While the best single feature S3 gave rise to 79.67% prediction strength with MCC = 0.5922 and AUC = 0.8835, the classifier using all the 20 features achieved only 75.45% prediction strength with MCC = 0.5791 and AUC = 0.8690. The possible explanation is that some of the 20 features contain redundant or correlated information, which may cause classifier performance degradation.

**Table 3:** Predictive performance of classifiers constructed by combining the best single features.

Features	AC (%)	SN (%)	SP (%)	ST (%)	MCC	ROC AUC
S3	82.53	72.19	87.15	79.67	0.5922	0.8835

S3, Bu	83.41	68.00	90.30	79.15	0.6019	0.8821

S3, Bu, Tt	82.88	61.90	92.26	77.08	0.5822	0.8725

S3, Bu, Tt, B	83.65	62.10	93.28	77.69	0.6009	0.8768

S3, Bu, Tt, B, Aa	83.65	61.90	93.36	77.63	0.6009	0.8743

S3, Bu, Tt, B, Aa, Mc	83.59	61.71	93.36	77.54	0.5993	0.8737

All 20 features	82.88	56.00	94.89	75.45	0.5791	0.8690

We then constructed SVM classifiers by combining some of the best single features for input encoding. Interestingly, none of these feature combinations gave rise to better classifier performance than the best single feature S3 (Table 3). For example, the classifier constructed using the best six single features (S3, Bu, Tt, B, Aa, and Mc) achieved only 77.54% prediction strength with MCC = 0.5993 and AUC = 0.8737.

To determine whether any combinations of the sequence features could improve classifier performance, we performed a brute-force search for the optimal feature subset. As shown in Table 4, classifier performance based on AUC was improved slightly but steadily when more features were used for input encoding. Among all the two-feature combinations, the biochemical feature Co (overall amino acid composition) together with the structural feature Bu (bulkiness) achieved the best classifier performance based on AUC (0.8872) with 80.54% prediction strength and MCC = 0.6057. These performance measures are slightly better than those of the empirical feature S3, a protein stability scale based on experimental data [17] . Significantly, the feature Co is also included in all the other feature subsets shown in Table 4, suggesting that the overall amino acid composition is highly relevant for sequence-based prediction of protein stability changes. For instance, the best four-feature subset contains the biochemical features Co and H (hydrophobicity index), the structural feature B (conformational parameter for beta-sheet), and the empirical feature S3. The classifier achieved 80.16% prediction strength with MCC = 0.6231 and AUC = 0.8940 (Table 4).

**Table 4:** Predictive performance of classifiers constructed using the optimal subsets of sequence features.

Features	AC (%)	SN (%)	SP (%)	ST (%)	MCC	ROC AUC
S3	82.53	72.19	87.15	79.67	0.5922	0.8835

Bu, Co	83.00	74.10	86.98	80.54	0.6057	0.8872

B, Co, S3	84.12	69.33	90.72	80.03	0.6194	0.8924

B, Co, H, S3	84.29	69.33	90.98	80.16	0.6231	0.8940

A, Aa, B, Co, P	84.47	70.48	90.72	80.60	0.6287	0.8954

A, Aa, B, Co, No, P	84.59	70.29	90.98	80.63	0.6310	0.8961

**Figure 3:**
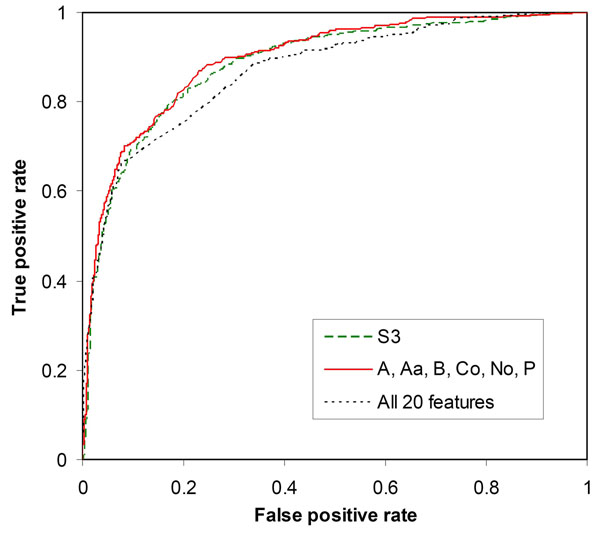
ROC curves for sequence-based prediction of protein stability change using multiple sequence features.

As shown in Table 4, the highest performance measures were obtained by using the optimal subset of six features, including the biochemical features Co and P (polarity), the structural features A (conformational parameter for alpha-helix), B and Aa (average area buried on transfer from standard state to folded protein), and the other biological feature No (number of codons for an amino acid). Classifier performance was not further improved significantly by including additional sequence features (data not shown). Interestingly, the optimal feature subset did not include the best single feature S3. The classifier constructed using the optimal feature subset achieved 80.63% prediction strength with MCC = 0.6310 and AUC = 0.8961. In Figure 3, this classifier’s ROC curve is compared with those of two other classifiers, one constructed using the best single feature S3, and the other trained with all the 20 features.

The results suggest that classifier performance can be enhanced by combining certain sequence features for input encoding. The optimal six-feature subset contains sequence features from different classes, especially biochemical features and structural features. Each of these features may not be an accurate scale of protein stability, but when combined, they can outperform the best empirical feature (S3) for predicting protein stability changes upon amino acid substitutions.

### Web server description

To make the accurate SVM classifier accessible to the biological research community, we have developed the MuStab web server (http://bioinfo.ggc.org/mustab/). Users can enter an amino acid sequence in FASTA format, and specify the position and the identity of the substituting residue. The system encodes the input sequence with the optimal feature subset, and then calls the svm_classify program of the SVMlight software package to classify the protein stability changes upon the amino acid substitution using the best SVM model developed in this study.

**Figure 4:**
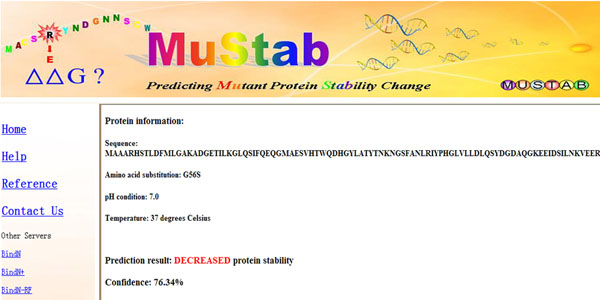
Sample output from the MuStab web server.

The output report returned from the MuStab web server includes the information about the query sequence and amino acid substitution, the prediction result, and the prediction confidence. The prediction result indicates either decreased or increased protein stability. The prediction confidence is based on the SVM output and computed as (1 – *s*), where *s* is the expected sensitivity for positive predictions or the expected specificity for negative predictions if the SVM output is used as the threshold in the ROC analysis (Figure 3). An example output report returned from the MuStab web server is shown in Figure 4 for the G56S mutation in spermine synthase (PDB: 3C6K), which causes X-linked Snyder-Robinson syndrome [28] . The amino acid substitution is predicted to decrease protein stability, and the prediction confidence is 76.34%.

## Conclusions

In this study, we have developed a machine learning method for predicting protein stability changes upon amino acid substitutions. The novelty of our method lies in the use of sequence features representing biological knowledge for input encoding. Twenty sequence features were examined for SVM classifier construction, and several of them were shown to be highly relevant for protein stability prediction. However, the SVM classifier constructed using all the twenty features did not show high predictive performance. We thus used a wrapper approach for feature selection, and identified the optimal subset of six sequence features for input encoding. The best classifier achieved the overall accuracy of 84.59% with 70.29% sensitivity and 90.98% specificity. This SVM classifier is compared favourably in performance with the previously published models for protein stability prediction. Since the previous studies did not utilize the biological knowledge for classifier construction, our method can be used to complement the existing methods to predict the consequences of amino acid alterations in disease candidate genes and may provide useful information for elucidating the molecular mechanisms of human genetic disorders. We have thus developed the MuStab web server (http://bioinfo.ggc.org/mustab/) to make our classifier accessible to the genetics research community.

## Competing interests

The authors declare that they have no competing interests.

## Authors’ contributions

LW and AKS initiated the project. LW and ST designed the study. ST conducted the data analysis, and drafted the manuscript. LW and AKS contributed to the manuscript preparation.
